# Impact of Genomic and Transcriptomic Resources on Apiaceae Crop Breeding Strategies

**DOI:** 10.3390/ijms22189713

**Published:** 2021-09-08

**Authors:** Fabio Palumbo, Alessandro Vannozzi, Gianni Barcaccia

**Affiliations:** Department of Agronomy Food Natural Resources Animals Environment, Campus of Agripolis, University of Padova, 35020 Legnaro, Italy; alessandro.vannozzi@unipd.it (A.V.); gianni.barcaccia@unipd.it (G.B.)

**Keywords:** marker-assisted selection, marker-assisted breeding, SNP variants, SSR markers, genome assembly, RNA-seq, reproductive barriers, cytoplasmic male-sterility, nuclear male-sterility, super barcoding

## Abstract

The Apiaceae taxon is one of the most important families of flowering plants and includes thousands of species used for food, flavoring, fragrance, medical and industrial purposes. This study had the specific intent of reviewing the main genomics and transcriptomic data available for this family and their use for the constitution of new varieties. This was achieved starting from the description of the main reproductive systems and barriers, with particular reference to cytoplasmic (CMS) and nuclear (NMS) male sterility. We found that CMS and NMS systems have been discovered and successfully exploited for the development of varieties only in *Foeniculum vulgare*, *Daucus carota*, *Apium graveolens* and *Pastinaca sativa;* whereas, strategies to limit self-pollination have been poorly considered. Since the constitution of new varieties benefits from the synergistic use of marker-assisted breeding in combination with conventional breeding schemes, we also analyzed and discussed the available SNP and SSR marker datasets (20 species) and genomes (8 species). Furthermore, the RNA-seq studies aimed at elucidating key pathways in stress tolerance or biosynthesis of the metabolites of interest were limited and proportional to the economic weight of each species. Finally, by aligning 53 plastid genomes from as many species as possible, we demonstrated the precision offered by the super barcoding approach to reconstruct the phylogenetic relationships of Apiaceae species. Overall, despite the impressive size of this family, we documented an evident lack of molecular data, especially because genomic and transcriptomic resources are circumscribed to a small number of species. We believe that our contribution can help future studies aimed at developing molecular tools for boosting breeding programs in crop plants of the Apiaceae family.

## 1. General Introduction

The Apiaceae family (previously known as Umbelliferae) is a nearly cosmopolitan taxon that includes about 3820 species organized into 466 genera [1]. According to the most recent and comprehensive phylogenetic study based on the analysis of the plastid *rpl16* intron and *trnD-trnY-trnE-trnT* intergenic spacers in 144 genera, the Apiaceae family is the youngest within the Apiales order and originated in Australasia ~87 Ma [2]. Apiaceae species are adapted to a wide range of conditions (from rocky to aquatic environments) and altitudes (from sea level to high mountains) but are predominant in temperate regions of Eurasia and North America and relatively rare in the humid tropics.

The family is organized into four main subfamilies, of which the Apioideae is the largest (2900 species and more than 400 genera [3,4]). The remaining species are distributed in the Azorelloideae, Mackinlayoideae and Saniculoideae subfamilies or remain unclassified (approximately 200 species [5,6]). Despite the presence of few large genera, each comprising from 130 to 250 species (such as *Eryngium*, *Bupleurum*, *Ferula* and *Seseli* genera), 85% is constituted by no more than ten species, and 25% by a single species (monospecific genera) [1].

Several species (almost exclusively from the Apioideae subfamily) are exploited for a wide range of purposes; the most important are summarized and referenced in Table 1.

Many umbellifers are consumed as food, where the edible part is constituted by the root (e.g., *Daucus carota*), the leaf (e.g., *Apium graveolens*) or the seed (e.g., *Cuminum cyminum*). It is worth underlining how several species were widely consumed in the past, while they have now been abandoned, replaced by more common species, or confined to a niche cuisine. This is the case of *Smyrnium olusatrum* (commonly known as Alexanders), whose flavor is intermediate between celery and parsley and was greatly appreciated by Roman and medieval cuisines but has been progressively replaced by celery [9]. For a comprehensive review of the nutraceutical properties of the main Apiaceae species, see [22]. Some Apiaceae find application in the beverage industry, where they are used mainly as flavoring (e.g., *Pimpinella anisum* and *Angelica archangelica*, respectively employed for the preparation of Greek ouzo and Italian vermouth). Several species are used as laxatives, diuretics, stimulants or sedatives; meanwhile, several species are established remedies or traditional/folk therapies against conditions, including spasmodic gastro-intestinal pains, digestive disorders, inflammations of mucous membranes of the upper respiratory tract and chronic cough [1,13,14]. Many experimental and biological investigations have been conducted to validate the antimicrobial and antifungal activities traditionally attributed to several Apiaceae species, such as *Carum carvi*, *Foeniculum vulgare*, *Coriandrum sativum* and *Pimpinella anisum* (for a review, see [13]). Some genera are widely used as ornamental garden plants (i.e., *Heracleum* spp., *Angelica* spp., *Astrantia* spp.), whereas some others are currently exploited for industrial purposes in the cosmetics sector (*Coriandrum sativum*, *Anethum graveolens*) or for the production of gums and resins (*Ferula* spp., *Dorema ammoniacum*). Finally, it is worth noting that not all Apiaceae species are edible; among the most toxic taxa are *Conium* spp. (poison hemlock), *Cicuta* spp. (water hemlock) and *Aethusa cynapium* (poison parsley). Despite their toxicity, the insecticidal activity of *Cicuta virosa* [23] and the analgesic and anti-inflammatory activity of *Conium maculatum* [24] represent some possible applications.

Global production statistics related to Apiaceae are scarce. The only two groups for which production data are available within the Food and Agriculture Organization of the United Nations (FAO) database are “carrot and turnip” and “anise, badian, fennel and coriander”. The estimated production of turnips (Brassicaceae) is less than 2% (US) and 1% (Europe) for carrots [25]. However, the production in the last 50 years (1969–2019) has more than quintupled to ca. 44 million tonnes [26], whilst the estimated Gross Production Value (GPV) is nearly USD 15 billion. In regards to the second group—where badian is the only non-apiaceous crop—in 50 years, the production grew about 20 times and is now ca. 1.9 million tons [26] with an aggregated GPV of USD 3.3 billion in 2018. The lack of production data for most of the Apiaceae species can be ascribed to the gap existing between few staple crops (e.g., carrot, fennel, celery, anise and coriander) produced on a large scale through robust breeding programs [8], while the remaining species are often confined to local production or collected from the wild [1], making it difficult to assess their economic value.

The economic influence of a species is a pivotal force driving the development of new relevant knowledge. Indeed, the information available for economically relevant species is expected to be far more abundant than the data available for species of little interest. To this end, the present review aims to highlight both the lack and progress achieved in the genomics and transcriptomics of this family, with a particular emphasis on their applications for breeding purposes.

## 2. Plant Reproductive Strategies and Breeding Schemes in the Apiaceae Family

Among the Apiaceae species exploited for food, medical, ornamental or industrial purposes, only a few are cultivated on a large scale following robust breeding strategies. Since a comprehensive understanding of reproductive biology is crucial for the success of a breeding plan, in this section, we will review the main reproductive strategies characterizing the Apiaceae family, along with any available information related to male sterility and self-incompatibility cases. Breeding strategies will also be discussed.

### 2.1. Plant Reproductive Systems in the Apiaceae Family

Apiaceae species reproduce sexually and are characterized by a common floral structure (simple or compound umbel); they also exhibit different crossing strategies because of the wide variation in the mode of pollination. Few cases of obligate outcrossing (i.e., strict allogamy) are known in dioecious (such as the Australian genera *Aciphylla* and *Anisotome*) and gynodioecious (i.e., female and hermaphroditic plants coexist, such as *Gingidia*, *Scandia* and *Lignocarpa*) taxa. An opposite situation is instead represented by the completely self-pollinated genus *Scandix* (to which the *S. pecten-veneris* leafy edible species belongs), where the anthers dehiscence occurs immediately above the stigma [27]. Beyond these extreme cases, Apiaceae exhibits a combination of allogamous and autogamous behaviors with a pollination that is almost exclusively insect-dependent. In this case, plants are generally hermaphrodite (bisexual flowers), although several cases of andromonoecious plants (i.e., hermaphrodite flowers coexisting with male flowers on the same plant) are known, even in core species such as *Daucus carota* [27,28], *Coriandrum sativum* [29] and *Pastinaca sativa* [30]. In particular, it has been shown that the number of hermaphrodite flowers decreases progressively from the first-order umbel to the last (for information on umbel orders, see Figure S1), while male flower numbers follow an opposite path, decreasing from the last (where it could be up to 100%) to the first order umbel [31]. Pollen produced by male flowers is mainly moved from pollinators to other plants, and only a small amount is used to pollinate hermaphrodite flowers of the same plants.

Although most of the Apiaceae are capable of both cross and self-fertilization, proterandry (male reproductive tissues maturate before females ones) and, to a lesser extent, protogyny (female reproductive tissues maturate before males ones) are frequently detected, thus significantly reducing the possibility of self-pollination [27]. Another factor that seems to discourage self-fertilization is the strong inbreeding depression observed both in economically important species, such as carrot [32], cumin [33], fennel [34] and coriander [35], as well as wild species such as *Eryngium alpinum* [36]. In the next section, the main reproductive barriers available in the Apiaceae family are discussed, along with possible applications for breeding purposes.

### 2.2. Using Plant Reproductive Barriers for Varietal Constitution

Self-incompatibility (SI) and male sterility (MS) represent the two most effective reproductive strategies to drastically reduce the occurrence of self-pollination. No cases of self-incompatibility have ever been reported in this family, except for a partial SI hypothesized for *Eryngium alpinum* and *Zizia* spp. [37]. The absence of known SI systems in the Apiaceae family (and many other families) gave rise to the hypothesis that the combination of proterandry/protogyny (defined as “temporal dioecism”) and andromonoecism—two characteristics widely spread in this family—may represent an evolutionary alternative to self-incompatibility [38].

On the contrary, MS seems to be more common within the Apiaceae family. Cytoplasmic male sterility (CMS), a maternally inherited trait that is often associated with single-gene mutations in the mitochondrial genome [39], has been reported in *Daucus carota* (for a review see [40]) but also in *Foeniculum vulgare* [41], *Apium graveolens* [42] and *Pastinaca sativa* [43]. In *Apium graveolens*, the discovery of CMS genotypes has been reported since the 1980s, and few F1 hybrids available on the market for celery and celeriac (*A. graveolens* var. *rapaceum*) were obtained using this reproductive barrier. However, information on breeding schemes is extremely vague [42,44]. On the contrary, CMS mutants have been available and extensively exploited since the 1940s, and the most widely grown carrot cultivars are nowadays hybrids. Of the two types of CMS identified in carrot, the ‘petaloid sterile’ is preferred over the ‘brown anther’ for hybrid development. In this latter type of CMS, first stamens form and appear phenotypically normal while complete microspore abortion and brown anthers turning brown are later observed. In ‘petaloid sterile’ plants, the anthers are transformed into petaloid structures during their early development and do not produce pollen [40]. In *Foeniculum vulgare* and *Pastinaca sativa*, the existence of CMS is documented [43,45,46], and several F1 hybrids are available on the market, but also, in this case, breeding data are quite scanty.

Due to the economic interest behind this trait, the mechanisms and origin of male sterility are often contained by the breeding companies, and reports on CMS sources are elusive. Only in few cases are the genetics underlying the lack of pollen elucidated [40,41]. It is well established that one of the most effective strategies to identify the genes responsible for CMS is the comparison between mitochondrial genomes (mtDNA) of fertile and sterile genotypes to identify differences in terms of sequences and/or structure organization. On the one hand, this is facilitated by the use of second- and third-generation sequencing platforms; meanwhile, on the other hand, there are still obstacles related to the structure of mtDNA. mtDNA assembly is deeply affected by the occurrence of repeated regions, nuclear and plastid-deriving sequences, heteroplasmy phenomena (co-existence of mitotypes) and several possible configurations (linear, circular or branched) [47,48]. This would explain why there are only two mitochondrial genomes available in NCBI for this family (i.e., *Daucus carota* and *Bupleurum falcatum*).

As previously introduced, the availability of CMS has facilitated the development of F1 hybrids for the exploitation of heterotic vigor. In a CMS-based hybrid production system, three lines are used: a CMS seed line (strain A), a male fertile sister line (also known as maintainer line, strain B) and a pollinator line (strain C) with a general combining ability (GCA) with the CMS line. Strain B and C are usually obtained through several generations of inbreeding to achieve the desired uniformity and homozygosity. The alternative to developing uniform, true-breeding lines are represented by the doubled haploid (DH) methodology, which allows the production of completely homozygous pure lines in less than two years. In *C. carvi*, DH plants have been constituted via anther culturing [49], while recently, a protocol for generating DH individuals starting from isolated microspore culture has been successfully applied in *Foeniculum vulgare*, *Anethum graveolens*, *Pimpinella anisum*, and *Carum carvi* [50].

**Figure 1:**
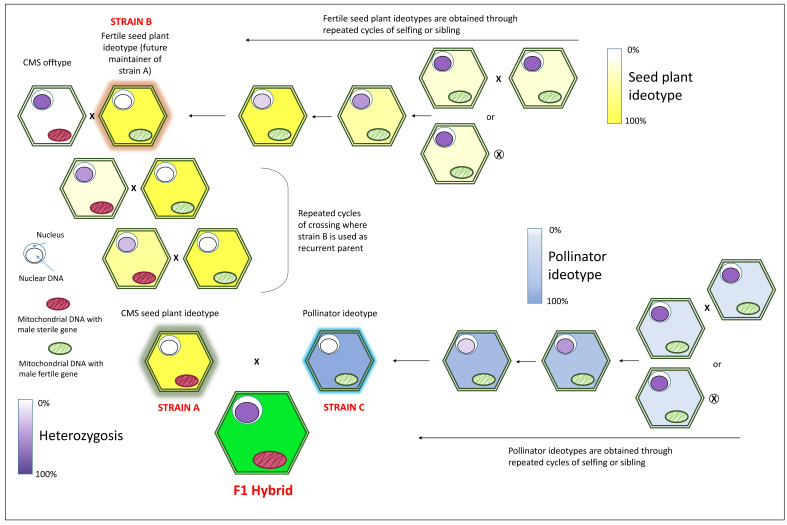
Simplified breeding scheme describing the development of F1 hybrids based on the exploitation of a cytoplasmic male sterility (CMS) system in combination with a maintainer line and an unrelated pollen donor or pollinator line. Three lines are used: a CMS seed line (strain A), a male fertile sister line (also known as maintainer line, strain B) and a pollinator line (strain C) with a general combining ability (GCA) with the CMS line. Strain B and C are initially obtained by means of several generations of selfing (⊗) or sibling (×) to achieve the desired ideotype (i.e., high uniformity and high homozygosis). The CMS seed line (strain A) is developed through backcross, using the newly constituted strain B (that acts as a recurrent parent) and a CMS genotype (used as a non-recurrent parent to transfer the male sterility trait into strain B). After several cycles of backcrossing (usually 6–7), the resulting progeny (strain A) will be isogenic to the B line except for the cytoplasm, which will be CMS. Strain B will finally serve as the maintainer of the newly obtained CMS seed plant.

The CMS seed line is instead obtained through a backcross program where a CMS genotype is used as a non-recurrent parent to introgress the male sterility trait into strain B, which acts as the recurrent parent. After several cycles (usually 6–7), the resulting progeny (strain A) will be isogenic to the B-line except for the cytoplasm, which will be CMS. The strain B will then serve to maintain the newly obtained CMS seed plant (Figure 1).

Inbreeding depression in Apiaceae (see previous section) represents one of the major obstacles encountered in the development of parent lines for F1 hybrid production. For example, in fennel, several generations of inbreeding negatively affected agro-morphological traits such as height, fresh and dry weight and number of fruit per umbel [34]. In this case, highly inbred parental lines can be used (as classically done in maize and pepper), but when the first signs of vigor-loss appear, it is necessary to intervene with hybridization cycles with related individuals [51]. Alternatively, instead of using a single combination of parent lines (strain A × strain C), a pool of homogeneous nearly isogenic parent lines is used, with an inevitable reduction in the uniformity of the resulting F1 hybrids.

When a nuclear gene (called restorer gene) is able to restore fertility in CMS lines, this reproductive barrier assumes the name of cytoplasmic-genic male sterility (CGMS). So far, CGMS has only been detected and exploited for F1 hybrid production in carrots [52].

**Figure 2:**
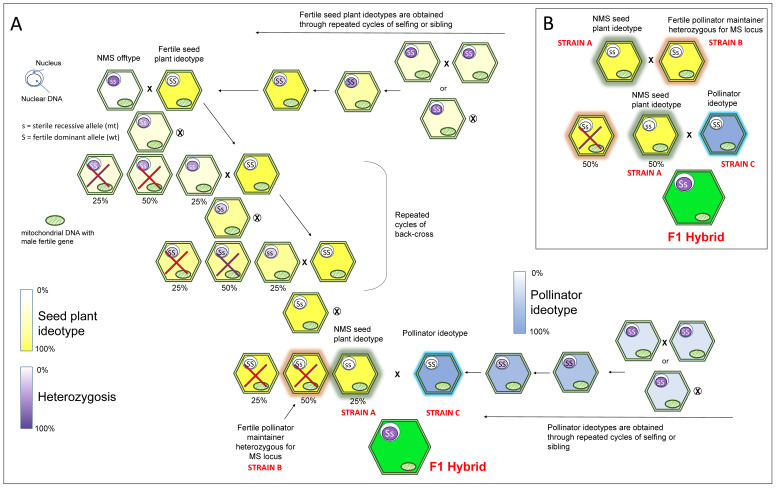
Simplified breeding scheme describing the development of F1 hybrids through the exploitation of a genic or nuclear male sterility (GMS or NMS) system relying on a single recessive nuclear gene(s). Three lines are used: a CMS seed line (strain A), a male fertile sister line (strain B, known as maintainer line of strain A) and a pollinator line (strain C) with a general combining ability (GCA) with strain A. (**A**) Fertile seed plants and strain C (pollinator ideotype) are subject to several generations of selfing (⊗) or sibling (×) to achieve the desired ideotype (i.e., high uniformity and high homozygosis). The NMS seed line (strain A) is produced by alternating cycles of backcross (ss sterile genotype × SS fertile seed plant, where this latter acts as a recurrent parent) and cycles of selfing/sibling between the Ss offspring resulting from the backcross. Only ss individuals (25%) are used in a new cycle of backcross. After several cycles, ss seed plant ideotypes (NMS) represent strain A (used for F1 hybrid production), whilst Ss individuals serve as a maintainer (strain B). (**B**) Strain A maintenance is achieved through a backcross with strain B: the resulting 50% of male fertile segregants (Ss) are identified and removed whilst the 50% male sterile (ss) are used as seed plants for F1 hybrid production.

Nuclear or genic male sterility (NMS or GMS) is the result of a single recessive nuclear gene(s) (although a dominantly inherited pattern is also possible). In this case, while pollinator lines (strain C) are classically obtained through several generations of inbreeding, the NMS seed line (strain A) is obtained by alternating cycles of backcross (ss sterile genotype × SS fertile seed plant ideotype, where this latter acts as a recurrent parent) and cycles of selfing/sibling between the Ss offspring resulting from the backcross. Only ss individuals (25%) will be then used for a new cycle of backcross (Figure 2A).

The maintenance of the NMS is tedious and performed through a backcross with an isogenic Ss line (strain B); the resulting 50% of male fertile segregants (Ss) need to be identified and removed (Figure 2B). NMS has been described in *Apium graveolens* [42] and *Daucus carota* [53], but to date, the use of this type of male sterility for commercial purposes in Apiaceae is scarcely documented. In fact, when both CMS and NMS male sterility systems are available, such as in the case of carrot and celery, the first is far more convenient because of its inheritance, mode of preservation and restoration of fertility [41,53].

Based on the most recent literature review, for other important crops such as *P. crispum*, *C. carvi*, *C. cyminum*, and *C. sativum*, no source for MS has been found yet, making the establishment of F1 hybrid lines impossible.

Intra- and inter-genera hybridizations have the potential to compensate for these gaps. CMS species A (CMS donor) can be used as a seed plant, while species B (CMS recipient) is used as a pollinator. The resulting hybrid (CMS), if able to produce female gametes, will act as a seed plant in backcross cycles with species B. With the exception of few successful inter-genera hybrids obtained by crossing *Apium* × *Petroselinum* [54], and several intra-genus wild [55] or experimental hybrids [56] within the *Daucus* genus, this type of study is substantially absent.

Emasculation and manual fertilization could represent possible strategies used to promote cross-pollination, but umbel size and morphology make stamen removal extremely complicated and time-consuming on a large scale. Thus, the use of manual pollination is limited to experimental purposes.

Male gametocide is an alternative to hand emasculation, but to date, few and not very effective experiments have been performed, only in *C. sativum* [57].

A biotechnological attempt to transfer CMS systems from one species to another could be made by asymmetric protoplast fusion. Recently, to develop alternative CMS lines, an asymmetric protoplast fusion was accomplished in celeriac: carrot, coriander and celery mesophyll protoplasts were used as a donor for protoplast fusion experiments while celeriac suspension cell protoplasts were used as acceptors. The constitution of viable alloplasmic hybrids, although at a very low frequency, was demonstrated [58]. It is clear that for the success of this methodology, an efficient protocol for protoplast regeneration is a pivotal starting point. So far, such protocols have been extensively studied only in carrot and celery [59,60].

Since CMS systems are not available in several Apiaceae species, the constitution of synthetic varieties represents an alternative breeding method. This method is based on the selection (based on GCA) and maintenance of a restricted number of parents that will be used for uncontrolled open pollination. Seeds from this crossing (Syn1), or further propagated seeds (Syn2-x), constitute a synthetic variety. This strategy is effectively used in *C. carvi* [61]. For some other economically important crops, such as *C. sativum* and *P. crispum*, cyclic breeding systems for the improvement of complex traits, where superior genotypes are identified and intercrossed to produce the next generation (e.g., recurrent selection and mass selection), are still applied [57,62].

Whatever the breeding scheme used, the selection of parental lines and the evaluation of the resulting offspring play a key role. This is pursued through the combined use of phenotypic and genotypic analyses.

## 3. Genomic and Transcriptomic Resources for Breeding Varieties and Phylogenetic Analyses

### 3.1. Whole Genome Sequencing Provides Powerful Tools for Marker-Assisted Breeding

The constitution of new varieties benefits from the synergistic effect of marker-assisted breeding (MAB) techniques and conventional breeding programs. The use of molecular markers is a well-established practice to estimate the homozygosity and to genotype both the parents (to be crossed) and the resulting offspring [46,63]. Markers are also widely used for the evaluation of DUS parameters (diversity, uniformity and stability) in the phase of variety constitution [64], for the legal registration of new varieties [65] and as an effective tool for addressing legal disputes related to improper use of registered varieties [66].

For this wide range of purposes, SNPs and SSR are the two most attractive classes of markers because of their reproducibility, co-dominant nature, locus-specificity and random genome-wide distribution.

Except for *D. carota*, whose economic importance led to the identification and development of thousands of SSRs and SNPs organized into more than 20 linkage maps [67], the amount of molecular markers available for the Apiaceae family is quite limited, especially concerning SNPs. In fact, the identification of this class of markers relies almost exclusively on sequencing, with costs that still represent a major obstacle for large-scale use in minor crops. However, SNP usage has exponentially increased over the past decade, thanks to the advent of next-generation sequencing (NGS) platforms and high-throughput techniques such as genotyping by sequencing (GBS) and double digest restriction-site associated DNA sequencing (ddRAD-seq). Furthermore, RNA-seq analyses have recently become one of the most representative genotyping techniques for the discovery of thousands of SNPs within coding regions at a reasonable cost. Most of the SNP-based studies performed within the Apiaceae family were mainly aimed at safeguarding or reconstructing the phylogenetic relationship and the geographical origin of species (e.g., *D. carota* [68,69,70], *Erigenia bulbosa* [71]) or tribes (e.g., Scandiceae [72]). With the exception of few cases, namely *Carum carvi* [73], *D. carota* [74] and *Foeniculum vulgare* [75], the use of SNPs for breeding purposes remains limited.

In comparison, the development and use of SSR markers within the Apiaceae family have made great strides. The higher polymorphic information content (PIC) and the possibility to transfer SSR among species or genera represent two of the most important advantages of SSRs over SNPs, which have ensured a greater diffusion. Until a few years ago, carrot was the only apiaceous plant for which a genome assembly [76] and robust set of SSRs [77] were available; the most common procedure was to assess the SSR primer transferability from carrot to other species of the same family. In this regard, transferability of SSR markers (i.e., through heterologous primers) was evaluated in other *Daucus* species as well as in *F. vulgare*, *Apium graveolens*, *Trachyspermum ammi*, *Coriandrum sativum*, *Pimpinella anisum*, *Cuminum cyminum* and *Anethum graveolens* [78,79,80,81], with a success rate ranging from 89% (within *Daucus* genus [81]) to 23% (for *F. vulgare* [78]). Overall, these studies highlight an extremely variable transferability rate highly associated with phylogenetic relatedness between the species. Given the limited success of the cross-genera transferability approach, alternative methodologies were employed in parallel for the development of microsatellites. Among them, biotinylated SSR primers were used for *Arracacia xanthorrhiza* [82], I-SSR in *Bupleurum chinense* [83], ddRAD-seq in *Heracleum* spp. [84] and data mining from EST-databases in *Centella asiatica* [85]. Moreover, RNA-seq analyses were used for microsatellite development in *Coriandrum sativum*, *Notopterygium incisum*, *Foeniculum vulgare*, *Oenanthe javanica*, *Apium graveolens*, *Angelica biserrata* and *Angelica dahurica* [75,86,87,88,89,90,91]; whereas DNA-seq runs were performed with the same goal in *Notopterygium oviforme*, *Anethum sowa*, *Cuminum cyminum*, *Scaligeria lazica*, *Foeniculum vulgare* [46,92,93,94,95]. In Table 2, we sum up what was described so far by reporting the main SSR-set developed in the Apiaceae family.

RNA-seq or gDNA-seq analyses allowed the identification of a number of SSRs of several orders of magnitude higher than that obtainable with methods such as I-SSR, biotinylated primers or heterologous primers. Table 2 also highlights the total absence of SSR markers for species of agronomic interest such as *Carum carvi* and *Pastinaca sativa*. For others (e.g., *Pimpinella anisum*), the number of validated microsatellites available is particularly low and challenging to use for MAB analyses. Further studies are therefore necessary to make up for these shortcomings.

The use of the aforementioned techniques for the development of microsatellite markers has been progressively abandoned, as the drop in sequencing costs has made the direct assembly of entire genomes more convenient in terms of time and cost. To date, there are only eight genome assemblies available for the Apiaceae (summarized in Table 3), all belonging to the Apioideae subfamily, except for *Centella asiatica* (Mackinlayoideae subfamily).

Of these, four genomes are finely assembled into pseudomolecules (i.e., chromosomes, *Apium graveolens* [98], *Daucus carota* [76], *Centella asiatica* [99] and *Coriandrum sativum* [100]); whereas the others (*Angelica gigas*, *Oenanthe javanica*, *Bupleurum falcatum* and *Foeniculum vulgare*) were incomplete and/or assembled at the scaffold level [46,96,97,101]. While the latter were obtained exclusively through short reads sequencing, the first four resulted from a combination of second- and third-generation sequencing platforms, proving to be the most suitable approach for obtaining fully assembled, highly accurate genome sequences. Based on the genome assemblies, an attempt to collect, organize and integrate genomic, transcriptomic and metabolic data was made through the development of integrated databases. To this aim, CarrotDB [102], Coriander Genomics DB [103] and Celery DB [104] were produced.

**Figure 3:**
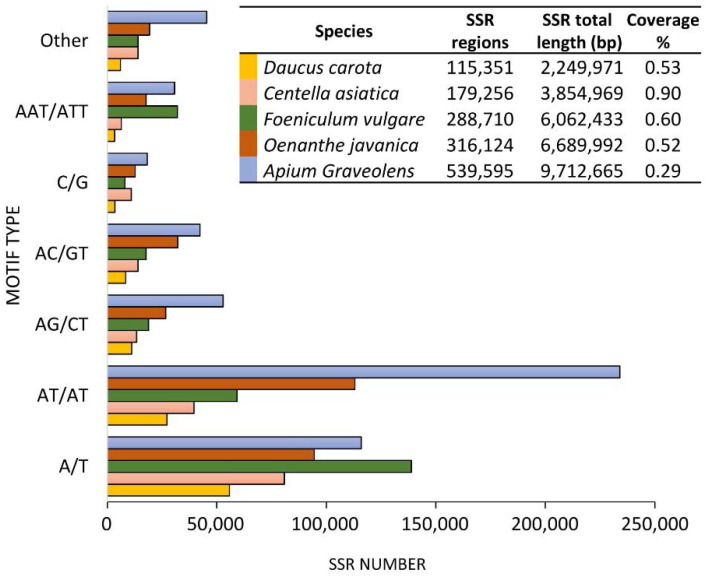
Distribution of the six most abundant microsatellite motifs in the five Apiaceae species for which genomes are publicly available. The table indicates the number of SSR regions identified in each genome, the total length (in bp) of the SSR regions, and the % of microsatellites on the overall genome size.

By using a predetermined PERL script (MIcroSAtellite, MISA [105]) on the five (of eight) genomes publicly available, we demonstrate the huge number of SSR markers that can be mined from a genome assembly and, therefore, the potential of this approach when compared to other techniques. The genomes from *Apium graveolens*, *Daucus carota*, *Centella asiatica*, *Oenanthe javanica* and *Foeniculum vulgare* were retrieved from NCBI and screened for mono-, di-, tri-, tetra-, penta- and hexanucleotide repeat motifs with a minimum repeat number of 10, 6, 5, 5, 5 and 5 respectively. Results are reported in Figure 3.

As expected, the SSR abundance was correlated with genome size since the species with the smallest (carrot 421 Mb) and largest (celery 3332 Mb) genomes showed, respectively, the lowest and highest numbers of SSR (in terms of SSR region and total length). On the other side, no correlation was observed between SSR coverage and genome size. These considerations should be taken with caution for *F. vulgare* and *O. javanica* as their genome assemblies are far from complete. Furthermore, since their assemblies relied solely on Illumina Sequencing, it should be emphasized that the exclusive use of short sequences can lead to an underestimation of the repeated regions that, in many cases, cannot be properly assembled (unless the repeated region is shorter than the read length) [106]. Of the two possible monomers, A/T is largely preferred to C/G in all the Apiaceae species analyzed, in agreement with what was recently highlighted by Srivastava et al. [107] in a meta-analysis performed on 71 plant species. Among the di-nucleotide motifs, AT/AT were more abundant than AG/CT and AC/GT, while AAT/ATT was the most represented tri-nucleotide motif. Both these findings were in agreement with Srivastava et al. [107]. By complementing the SSR survey results with dedicated software to design primers in batch (e.g., BatchPrimer3 [108]), it is possible to obtain dozens of SSR-specific primer couples to be tested and eventually used for MAB analyses.

Overall, complete genome assemblies reduce the effort and time required for conventional MAB and MAS approaches. In fact, they represent a treasure trove of markers to be exploited for MAB purposes and a valuable opportunity to decipher hundreds of functional and regulatory networks. Genome assemblies, supported by RNA-seq experiments, make it feasible to identify and characterize the genes controlling the agronomic traits of interest and tagging molecular markers to be used for introgression practices.

### 3.2. RNA-seq Analyses Are Starting Points for the Identification of Genes Responsible for Traits of Agronomic Interest

RNA-seq studies speed up the identification of genomic loci responsible for traits of agronomic interest. Several transcriptome profiling studies (sometimes supported by metabolic analyses) have been conducted to elucidate key pathways involved in stress tolerance, in the production of metabolites of interest and in plant growth and development. In any case, the progress of knowledge did not occur uniformly throughout the Apiaceae family but proportionally to the economic weight of each species.

In regard to the characterization of metabolites of agronomic and nutritional interest, carrots are a significant source of carotenoids and anthocyanins and most transcriptomic studies have been conducted on the edible part (root tissues) to identify genes, miRNA and transcription factors (TF) involved in the biosynthesis of these two major classes of compounds [109,110,111,112,113]. Among the TF involved in the anthocyanin pathway, *bHLH-A* (*HELIX-LOOP-HELIX PROTEIN A*), *TTG1* (*TRANSPARENT TESTA GLABRA 1*), *MYB6*, *MYB7*, *MYB3-like* and three *RAX2-like* genes (*REGULATOR OF AXILLARY MERISTEMS 2-like*) were found to be positively correlated with anthocyanin content, whilst *bHLH137-like* and two *FNS/F2H-like* genes (*FLAVONE SYNTHASE*/*2S-FLAVANONE-HYDROXYLASE-like*) act as downregulators [109,110,113]. *GST1* (*GLUTATHIONE S-TRANSFERASE 1*) is thought to be an important anthocyanin transporter, whose upregulation leads to a vacuolar anthocyanin accumulation [110]. By comparing the transcriptomic profiles of leaf and root, Ma et al. identified 15 main genes putatively involved in carotenoid accumulation, with particular reference to *PSY2* (*PHYTOENE SYNTHASE 2*), *PDS* (*PHYTOENE DESATURASE*), *CISO1* (*CAROTENOID ISOMERASE 1*), and *BCH1* (*β-CAROTENE HYDROXYLASE 1*) [112].

To a lesser extent, similar studies have also been carried out in other species to elucidate taxon-specific biosynthetic pathways. Since *Ferula gummosa* and *F. asafoetida* are highly recognized for their oleo-gum-resin products, commonly known as galbanum and constituted by different types of terpenes (myrcene, germacrene-D, α-terpineol, and β-amyrin). The biosynthetic pathways of these compounds were extensively investigated through RNA-seq analyses [114,115,116]. These studies led to the identification of four key genes, namely *α-TS* (*A-TERPINEOL SYNTHASE*), *MYS* (*MYRCENE SYNTHASE*), *GDS* (*GERMACRENE-D SYNTHASE*) and *β-AS* (*Β-AMYRIN SYNTHASE*), whose expression was particularly high in the roots. Similarly, the biosynthetic pathway of anethole, a phytoalexin with antimicrobials, antifungals and insecticidal properties, has been recently elucidated through the transcript profiling of *Foeniculum vulgare* [75]. Despite the identification of 11 putative genes involved in the biosynthesis of anethole/methyl chavicol, their expression in different tissues has never been investigated. An attempt to reveal the major enzymes leading to the production of thymol (a monoterpene compound well known for multiple biological activities against fungi, bacteria and viruses) was made in *Trachyspermum ammi* (Ajowan) [117]. In particular, *TPS1*, *TPS2* (*TERPENE SYNTHASE 1* and *2*), and two members from the dehydrogenase and the cytochrome P450 families, seem to be decisive for the production of this metabolite. The same species are inflorescence-rich in secondary metabolites, while another RNA-seq analysis was aimed at the identification of genes involved in terpenoid and brassinosteroid accumulation [118]. Coumarins and coumarin-derived compounds (e.g., furanocoumarins) play a repulsive and toxic role against certain insect larvae, and the key genes involved in their biosynthesis have been greatly investigated in *Pastinaca sativa* [119], *Angelica dahurica* [120] and *Apium graveolens* [98]. In particular, Munakata et al. identified two different prenyltransferases (*PT1* and *PT2*) responsible for the synthesis of the two furanocoumarin isomers (linear and angular) in *P. sativa* [119]; whilst Zhao et al. and Song et al. elucidated the seven enzymes leading to coumarin production in *A. dahurica* and *A. graveolens*, respectively [98,120]. Among the triterpene saponins employed in traditional medicine, saikosaponins and asiaticoside are particularly abundant in *Bupleurum chinense* [121] and *Centella asiatica* [122,123], and the main enzymes involved in their synthesis are currently under study. In *B. chinense*, two CYP450*s* (*P450-7* and *P450*-*12*) and three UGTs (*URIDINE DIPHOSPHATE GLYCOSYLTRANSFERASES 3, 5* and *15*) were identified as the most likely candidates involved in saikosaponin biosynthesis [121], whereas studies focused on the triterpene pathways led to the isolation of *UGT73AH1* (*ASIATIC ACID GLUCOSYLTRANSFERASE*), pivotal for asiaticoside biosynthesis in *C. asiatica* [122]. Transcriptome analysis conducted in *Coriandrum sativum* revealed 16 candidate genes controlling the biosynthesis of monoterpenoids, triterpenoids and sesquiterpenoids [88]. Of these, two terpene synthase candidate genes, *TRPS* (*TERPINENE SYNTHASE*) and *LINS* (*S-LINALOOL SYNTHASE*), were also characterized and expressed in bacteria, demonstrating their capability to catalyze the conversion of geranyl diphosphate (the precursor to monoterpenes), to c-terpinene and (S)-linalool, respectively [124]. In a second RNA-seq based study, ten tyrosine metabolic pathway-related genes (TMPRGs), six porphyrins and chlorophyll metabolic pathway-related genes (PCMPRGs), and five Vitamin E metabolic pathway-related genes (VEMPRGs) were identified in coriander [125]. Finally, four key genes, namely *ACPD1/3* (*ACYL CARRIER PROTEIN DESATURASE 1/3*), *KAS I-1* (*3-KETOACYL-ACP SYNTHETASE I-1*), *FATB-1/3* (*ACYL-ACP THIOESTERASE B*), and *DGAT2* (*DIACYLGLYCEROL ACYLTRANSFERASE 2*), putatively responsible for the accumulation of petroselinic acid, a monounsaturated fatty acid with many prospective applications in both functional foods and pharmaceutical industries, were identified in *C. sativum* [126].

Understanding the molecular mechanisms underlying plant growth and development is key to crop breeding, especially in relation to those tissues that embody the commercial product. For example, elucidating the mechanisms regulating root development and root biomass accumulation in *Bupleurum chinense* and *B. scorzonerifolium* is pivotal, considering their roots are widely used for the treatment of inflammatory disorders and infectious diseases. To this aim, an RNA-seq based study identified a subset of candidate genes involved in auxin signal transduction and explored their functions in lateral root development [127]. On the same path, Yu et al. showed that inflorescence removal (disbudding) is able to ameliorate primary root length, biomass accumulation and saikosaponin (SS) content by acting on brassinolide (BR) signal transduction, jasmonic acid (JA) signal transduction and SS pathways [128]. In celery, a transcriptomic study was performed to investigate the specific pathways involved in cellulose and pectin metabolism and, possibly, in the formation of the hollow or solid petiole, whose role is pivotal in sustaining the entire plant [91]. The same study also focused on genes that may affect early bolting, a premature (before harvesting) rapid elongation of the main stem into a floral axis that dramatically reduces the commercial quality of celery. In this case, *ELF7* and *ELF8* (*EARLY FLOWERING 7* and *8*), *FCA* (*FLOWERING TIME CONTROL*), *FLC* (*FLOWERING LOCUS C*), *TFL2* (*TERMINAL FLOWER 2*) and *PIE1* (*PHOTOPERIOD-INDEPENDENT EARLY FLOWERING 1*) were found to be key genes involved in the flowering process. Finally, since plant developmental processes, such as apical dominance, later root initiation and vascular differentiation, are greatly influenced by auxins, the auxin response factor (ARF) family has been investigated in celery, coriander and carrot, leading to the identification of 28, 34 and 27 genes, respectively [129].

A third line of studies was oriented towards the identification of key genes involved in response to biotic or abiotic stresses, mainly through comparative RNA-sequencing analysis of resistant/tolerant and susceptible genotypes. Among the major abiotic stresses, drought, high salinity and extreme temperatures can deeply affect the yield and quality of production; several studies have attempted to identify the biosynthetic pathways activated in response to these stresses. For example, in *Petroselinum crispum*, *Oenanthe javanica* and *Apium graveolens*, respectively, 7 AP2/ERF TF, 10 miRNA and 8 genes were found to be responsive to cold, heat, drought and salt stresses [87,130,131]. In *Notopterygium incisum*, 21 transcripts were related to cold tolerance [86] and among them, stand out the genes homologous to *COR47* (*COLD-REGULATED GENE*), *GIF2* (*GRF1-INTERACTING FACTOR 2*) *PGP20* (*P-GLYCOPROTEIN 20*), *RECQ4A* (a DNA helicase from the RecQ family) and *UGT80B1* (*UDP-GLYCOSYLTRANSFERASE 80B1*). In *Apium graveolens*, 24 genes associated with sulfur and selenium compound metabolism, including four ATP-sulfurylases, resulted in differential expression with selenium treatment [132]. Since heat is one of the most detrimental stresses, major efforts are underway to identify heat-responsive genes in several species. HSFs (heat shock transcription factors) are key TFs that respond to heat stress and play an important role in heat resistance. To this aim, by studying the expression of *HSF* family genes in celery, coriander, and carrot, Pei et al., identified 17, 32 and 14 (respectively) candidate genes whose role in heat tolerance could be decisive [133].

As for the biotic stresses, we mention the efforts made in *Coriandrum sativum* for the identification of genes able to confer resistance to stem gall disease [134,135] such as *USP* (*UNIVERSAL STRESS PROTEIN*), *ANK* (*ANKYRIN REPEAT-CONTAINING PROTEIN*), *PDR* (*PROBABLE DISEASE RESISTANCE*) and *HSP20* (*HEAT SHOCK PROTEIN 20*). A single recessive resistance gene (*cmv*) against Celery Mosaic Virus (CeMV), the most common viral disease of celery, was identified by D’Antonio et al. [136], and two markers (*me1em2* and *me8em2*) were found to be tightly associated with this locus [137].

It must be stressed that most of the studies performed within the Apiaceae family are still preliminary and mainly based on the discussion of differentially expressed genes (DEG). Functional studies performed on candidate genes putatively responsible for resistance are indeed extremely rare and available almost exclusively for *Daucus carota* and, to a lesser extent, *Apium graveolens*. In celery, most of the functional studies were aimed at deciphering the mechanisms of response to cold and salt stresses. In this regard, the overexpression of two celery dehydration-responsive-element-binding (DREB) TF (*AgDREB1* and *AgDREB2*) in Arabidopsis induced an increased tolerance to cold treatment [138] whilst a mannose-6-phosphate reductase (*M6PR*) coding gene [139] and two sucrose uptake transporter genes (*AgSUT1* and *AgSUT2*, [140]) resulted directly in salt tolerance. The mannitol pathway seems to also be involved in biotic stress resistances: transgenic tobacco plants constitutively expressing a celery mannitol dehydrogenase (*MTD*) acquired a significantly enhanced resistance to *Alternaria alternata* [141].

For *Daucus carota*, the situation is far more advanced—extensive studies were performed to select cultivars with partial or complete resistance to both biotic and abiotic stresses. Although the genetic basis of most of these resistances is yet to be determined, some resistance genes have already been identified and mapped in the carrot genome. For an exhaustive examination of the genetic and genomic advancements made against biotic and abiotic stress in carrots, we suggest, respectively, the reviews from du Toit et al. [142] and Grzebelus [143]. This wealth of information, together with the advancement of tissue culture research, favors the development of engineered carrot genotypes with improved traits. As summarized by Baranski and Lukasiewicz [144], carrot is the only species of this family for which genetic engineering techniques have been applied to introduce resistances against leaf (e.g., *Alternaria* spp., *Cercospora carotae*, *Erysiphe heraclei* and *Xanthomonas hortorum*) and root pathogens (e.g., *Sclerotinia sclerotiorum* and *Botrytis cinerea*). In the same way, enhanced tolerance to drought, salinity and herbicides were also achieved. Finally, since carrot is grown worldwide, it can be stored for a long period, can be eaten raw and represents an attractive candidate for the production of recombinant proteins to be used for the treatment of human diseases, including but not limited to diabetes, tuberculosis, hepatitis B and cholera [144]. However, no genetically modified carrots have ever been commercialized or included in pre-registration field trials.

### 3.3. From Gene to Genome: cpDNA Is Improving Phylogenetic Analyses through Super-Barcoding

**Figure 4:**
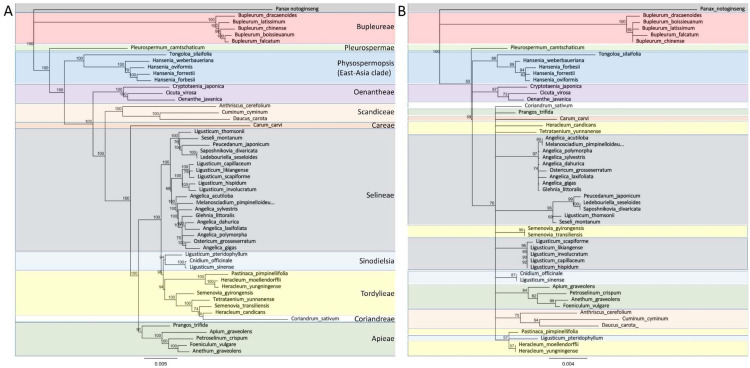
Phylogenetic relationships inferred from 53 species of the Apiaceae family by using *Panax notoginseng* (Arialaceae) as an outgroup; each clade is highlighted with a different color. (**A**) The 54 plastid genomes were retrieved from GenBank (Table S1) and aligned using MAFFT v7.450 [150] under default parameters; Geneious prime (Biomatters, Inc., San Diego, CA, USA) was used to reconstruct the phylogenetic relationships based on the Neighbor-Joining (NJ) method, and 1000 bootstrap replicates. (**B**) *rbcL* sequences were extracted from the 54 plastid genomes analyzed in Panel A and aligned using MAFFT v7.450 [150]; the phylogenetic relationships based on the Neighbor-Joining (NJ) method and 1000 bootstrap replicates were reconstructed using Geneious prime (Biomatters, Inc., San Diego, CA, USA) under default parameters.

Differently from nuclear (nuDNA) and mitochondrial (mtDNA) genomes, that find their application in the constitution of new varieties, chloroplast DNA (cpDNA) is useful for resolving systematic relationships among genera and species. For this purpose, the CBOL Plant Working Group [145] recommends the use of *rbcL* and *matK*, while additional informative loci are the intergenic spacer *trnH*-*psbA* and the *trnL* intron. However, as demonstrated by the most comprehensive DNA barcoding-based study performed in the Apiaceae family on 1957 species in 385 diverse genera [146], the identification to species level is not always feasible, even combining together all the abovementioned barcoding genes. Luckily, the development of NGS data exponentially increased the amount of data available, allowing the exploitation of entire chloroplast genomes (the so-called “super-barcodes” [147]) for phylogenetics. Few recent studies have evaluated the phylogenetic relationships among Apiaceae species based on their whole plastid genome alignments (e.g., [148]), while in the most comprehensive meta-analysis, Wen et al. [149] analyzed Apiaceae members based on 74 CDS concatenated sequences common to all species. Although the reconstruction of the phylogenetic relationships existing within this complex family is beyond the aim of this review, a super-barcode-based analysis was performed to demonstrate its capability to provide valuable information for more accurate systematic and evolutionary investigations when compared to a single-locus-based DNA barcoding analysis. To this aim, the 53 plastid genomes available from species of the Apiaceae family were retrieved from GenBank (Table S1). Their size ranges from 141.9 Kb (*Heracleum candicans*) to 164.7 Kb (*Peucedanum japonicum*) and contains a CDS number varying from 78 to 99. In addition, the cpDNA of *Panax notoginseng* was selected as the outgroup since its family (Araliaceae) is from the same order (Apiales) of Apiaceae. The 54 plastomes were aligned using MAFFT v7.450 [150] under default parameters, while Geneious prime (Biomatters, Inc, San Diego, CA, USA) was used to reconstruct the phylogenetic relationships based on the Neighbor-Joining (NJ) method with 1000 bootstrap replicates (Figure 4A).

With the same approach, we re-analyzed the same 54 species solely on the basis of the *rbcL* sequences extracted from the plastomes (Figure 4B). The *rbcL*-based tree shows uncertain relationships. In fact, except for Bupleureae, which experienced the earliest differentiation, and the groups Pleurospermeae, Physospermopsisa and Oenantheae, it was impossible to discriminate species from closely related tribes (e.g., Tordylieae and Selineae). On the contrary, within the plastome-based tree, all the species were correctly grouped into their 11 relative tribes with bootstrap values usually equal to 100. Moreover, with the exception of the Tordylieae tribe organization, the relationships existing among these 11 tribes matched with those reported by Wen et al. [149], despite the two analyses being conducted differently (i.e., aligning entire plastomes and aligning 74 concatenated CDS, respectively). In Wen et al., Tordylieae failed to form monophyly and resulted in a split into two subclades: Tordyliinae I (that included *Pastinaca* and *Heracleum* genera) as a sister group of Selineae, and Tordyliinae II (that included *Semenovia* and *Tetrataenium* genera) as the sister group of Coriandreae. On the contrary, in our whole plastome-based analysis, all the Tordylieae species seem to descend from a common ancestor. Regarding the tight clustering of *Coriandrum sativum* with the Tordylieae tribe, both analyses support the hypothesis that an early lineage of the Coriandreae tribe could have captured the chloroplast genome of the ancestors of Tordylieae [149]. Noteworthy is the confirmation of the complexity of the *Ligusticum* genus, as already demonstrated by a previous *ITS*-based study [151]. The eight *Ligusticum* species analyzed here were clustered in two groups: Sinodielsia and Selineae, in accordance with what was reported in the *ITS*-based study. Finally, peculiar are the results obtained for *Saposhnikovia divaricata* (NC_050292.1) and *Ledebouriella seseloides* (NC_034643.1), both from the Selinae tribe. Based on their alignment, they share 99.997% of their identity and only four polymorphic positions. From a literature review, we found out that these two species are extremely phylogenetically related [152]; in some cases, they are even considered synonyms of the same species [153]. Overall, taking advantage of the case study illustrated here, we stress that the resolution and precision offered by the comparison of complete plastid genomes in the reconstruction of the phylogenetic relationship clearly exceed the possibilities offered by single-gene analyses. The low numbers of plastomes available today represent the most important limit for the exploitation of super barcoding, but we are confident that the continuous decline in the cost of sequencing will lead to an exponential growth of available cpDNAs.

## 4. Concluding Remarks

The Apiaceae family includes thousands of species; nonetheless, the vast majority of the genomic and transcriptomic data available concern a limited number of crop plant species of economic interest, mainly *D. carota* and *A. graveolens*. Few cases of male sterility are known, and they are exploited for the production of commercial F1 hybrids, but the information is quite scanty and obviously contained by breeding companies. For species with no known cases of male sterility, there are multiple alternatives to avoid or limit self-pollination, but most of them have been poorly taken into account. This would explain why phenotypic-aided recurrent selection and mass selection-based breeding programs are still widely used in several apiaceous crops.

In the meta-analysis of the molecular markers available for marker-assisted breeding, the situation is slightly better even if for some crops of agricultural interest (e.g., *Carum carvi*, *Pastinaca sativa* and *Pimpinella anisum*), panels of markers supporting varietal constitution are absent or insufficient. Although whole-genome sequencing is one of the best approaches for identifying markers, assembled genomes (fully or partially) are only available for eight species. Even the studies related to the identification of genes involved in stress tolerance or in the production of metabolites of interest are, with the exception of *D. carota*, still in an embryonic state. In fact, although dozens of RNA-seq analyses have enabled the identification of hundreds of candidate genes, functional studies are almost completely absent. In the last section, we emphasized how cpDNA, although not directly involved in the varietal constitution (like nuDNA and mtDNA), represents the future of omics-based phylogenetic studies and is destined to replace single-gene-based DNA barcoding.

**Figure 5:**
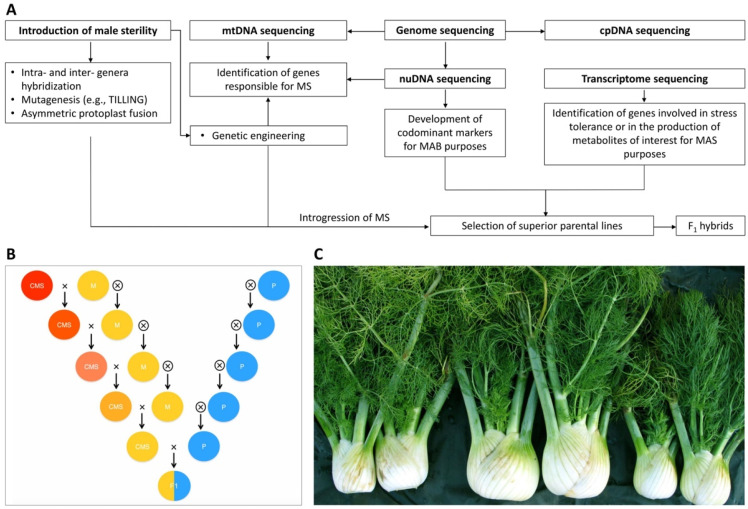
(**A**) Synoptic flowchart summarizing the impact of male sterility systems, genomic and transcriptomic resources on breeding strategies. (**B**) Breeding methods exploitable for the selection of CMS inbred lines morphological similar to maintainers (M) and genetically differentiated to pollinators (P) used for the development of F1 hybrids in fennel (×, sibling; ⊗, selfing). (**C**) Example of fennel commercial products of F1 hybrids (center) with parental plants, i.e., female male sterile inbred line (left) and male inbred line (right) (source: courtesy of Dr. Luca Pallottini).

Overall, based on the literature reviewed in this study, there emerges a close correlation between the existence of robust breeding programs for the constitution of F1 hybrid varieties and the availability of both reproductive mutants and molecular resources. The summary flow chart reported in Figure 5A illustrates how the development of markers through genome and transcriptome sequencing, together with the discovery or transfer of MS systems, contribute to the establishment of superior parental lines and F1 hybrids.

It is well established that the production of F1 hybrids in economically relevant species such as carrot, celery and fennel (Figure 5B,C) would not be possible without efficient CMS/NSM systems and, in the absence of molecular markers, be useful for both the characterization and selection of superior parental lines (MAB) and traits of agronomic interest (MAS).

We believe that the serious shortcomings pointed out in this study will encourage future studies aimed at the development of next-generation molecular tools to be exploited in crop plants whose agronomic improvements are still based exclusively on conventional and suboptimal breeding programs.

## Figures and Tables

**Table 1:** Main uses of Apiaceae species in the agrifood and biopharmaceutical industries.

Use	Examples	References
Food (root)	*Daucus carota*, *Pastinaca sativa*, *Apium graveolens* (var. *rapaceum*), *Arracacia xanthorrhiza*, *Bunium bulbocastanum*, *Cryptotaenia canadensis*, *Sium sisarum*	[1,7,8]
Food (leaf)	*Apium graveolens*, *Foeniculum vulgare*, *Pimpinella anisum*, *Coriandrum sativum*, *Petroselinum crispum*, *Angelica* spp., *Anthriscus cerefolium*, *Levisticum officinale*, *Smyrnium olusatrum*, *Centella asiatica*, *Oenanthe javanica*	[1,7,8,9]
Food (seed)	*Apium graveolens*, *Pimpinella anisum*, *Anethum graveolens* and *Foeniculum vulgare*, *Coriandrum sativum*, *Cuminum cyminum*, *Smyrnium olusatrum*	[1,7,8]
Beverages	*Pimpinella anisum* (for anisette, ouzo, and raki), *Carum carvi* (for akvavit and Kümmel), *Angelica archangelica* (for vermouth and Chartreuse)	[8,10,11,12]
Pharmacological use and folk remedies	*Foeniculum vulgare*, *Petroselinum crispum*, *Pimpinella anisum*, *Carum carvi*, *Visnaga daucoides*, *Centella asiatica*, *Cuminum cyminum*, *Pastinaca sativa*, *Apium graveolens*	[1,13,14]
Antibacterial and antifungal properties	*Cuminum cyminum*, *Carum carvi*, *Foeniculum vulgare*, *Coriandrum sativum*, *Pimpinella anisum*, *Anethum graveolens*, *Petroselinum crispum*, *Ferula* spp.	[13,15,16]
Ornamental plants	*Eryngium* spp., *Heracleum* spp., *Angelica* spp., *Astrantia* spp., *Bupleurum* spp.	[1]
Fragrance and cosmetics	*Coriandrum sativum*, *Anethum graveolens*	[13]
Gum and resins	*Ferula* spp., *Dorema ammoniacum*	[17,18]
Toxic	*Cicuta* spp., *Conium maculatum*, *Bupleurum* spp., *Aethusa cynapium*	[19,20,21]

**Table 2:** SSR (genomic SSR, gSSR or expressed sequence tag-derived SSR, EST-SSR) marker sets available for Apiaceae species along with the methodology employed for microsatellite identification and, when available (n.s. = not specified), the number of samples used for validation and the resulting Polymorphism Information Content (PIC) range.

Species	Methodology	SSR Identified	SSR Validated	Samples Tested	PIC	Ref
*Anethum graveolens*	SSR transfer from *D. carota*	30 gSSR	15 gSSR	5	n.s.	[80]
*Anethum sowa*	gDNA-seq	48,951 gSSR	10 gSSR	n.s.	n.s.	[94]
*Angelica biserrata*	RNA-seq	8371 EST_SSR	17 EST-SSR	208	0.44–0.83	[90]
*Angelica dahurica*	RNA-seq	33,724 EST-SSR	10 EST-SSR	56	0.27–0.63	[89]
*Angelica gigas*	gDNA-seq	138,113 gSSR	36 gSSR	16	0.44–0.89	[96]
*Apium graveolens*	RNA-seq	80 EST-SSR	28 EST-SSR	31	0.06–0.72	[91]
*Arracacia xanthorrhiza*	biotinylated SSR primer	26 gSSR	14 gSSR	58	0.00–0.65	[82]
*Bupleurum chinense*	I-SSR	100 gSSR	19 gSSR	22	0.20–0.92	[83]
*Bupleurum falcatum*	gDNA-seq	91,377 EST-SSR	21 gSSR	n.s.	n.s.	[97]
*Centella asiatica*	data mining from EST-db	686 EST-SSR	18 EST-SSR	n.s.	n.s.	[85]
*Coriandrum sativum*	RNA-seq	9746 EST-SSR	76 EST-SSR	14	0.00–0.79	[88]
*Cuminum cyminum*	gDNA-seq	8086 gSSR	23 gSSR	30	0.03–0.70	[95]
*Daucus carota*	hybridization-based library	n.s.	196 gSSR	n.s.	n.s.	[81]
*Foeniculum vulgare*	gDNA-seq	103,306 gSSR	27 SSR	100	0.03–0.92	[46]
*Heracleum* spp.	ddRAD-seq	54 gSSR	19 gSSR	48	n.s.	[84]
*Notopterygium incisum*	RNA-seq	13,149 EST-SSR	19 EST-SSR	24	0.53–0.83	[86]
*Notopterygium oviforme*	gDNA-seq	793	17 gSSR	94	0.37–0.64	[92]
*Oenanthe javanica*	RNA-seq	1233 EST-SSR	n.s.	n.s.	n.s.	[87]
*Pimpinella anisum*	SSR transfer from *D. carota*	30 gSSR	16 gSSR	5	n.s.	[80]
*Scaligeria lazica*	gDNA-seq	1982	40 gSSR	40	0.37–0.84	[93]

**Table 3:** Genome assemblies are available for the Apiaceae family, along with the common and scientific name, chromosome (Chr) number, estimated and assembled genome size, level of assembly (chromosome, Chr or Scaffold, Scaff), sequencing platform used and reference. Genomes publicly available are underlined. (n.s. = not specified).

Common Name	Scientific Name	Chr Number	Genome Size (Mbp)		Assembly Level	Sequencing Platforms	Ref
			Estimated	Assembled			
Celery	*Apium graveolens*	2*n* = 22	n.s.	3332	Chr	Illumina Hiseq4000; PacBio Seq I; Hi-C; 10x Genomics	[98]
Carrot	*Daucus carota*	2*n* = 18	473	421	Chr	Illumina Hiseq2000, Sanger (BAC libraries), 454 GS FLX	[76]
Asiatic pennywort	*Centella asiatica*	2*n* = 18	430	430	Chr	10x Genomics, Hi-C, Illumina Hiseq X	[99]
Coriander	*Coriandrum sativum*	2*n* = 22	2130	2119	Chr	Illumina Miseq, PacBio Seq I, 10x Genomics, Hi-C	[100]
Korean angelica	*Angelica gigas*	2*n* = 22	2670	804	Scaff	Illumina Hiseq2500	[96]
Java waterdropwort	*Oenanthe javanica*	2*n* = 22	n.s.	1278	Scaff	Illumina Hiseq2500	[101]
Sickle hare’s-ear	*Bupleurum falcatum*	2*n* = 16	2120	922	Scaff	Illumina Hiseq2000	[97]
Fennel	*Foeniculum vulgare*	2*n* = 22	1320	1010	Scaff	Illumina Hiseq2500	[46]

## Data Availability

The data presented in this study are available within the article or as supplementary materials.

## Supplementary Materials

The following are available online at https://www.mdpi.com/article/10.3390/ijms22189713/s1.
