# Supplementary figures and images for: Impact of Genomic and Transcriptomic Resources on Apiaceae Crop Breeding Strategies
Source: Int J Mol Sci. 2021 Sep 8;22(18):9713. doi: 10.3390/ijms22189713 (PMC8465131; doi:10.3390/ijms22189713)

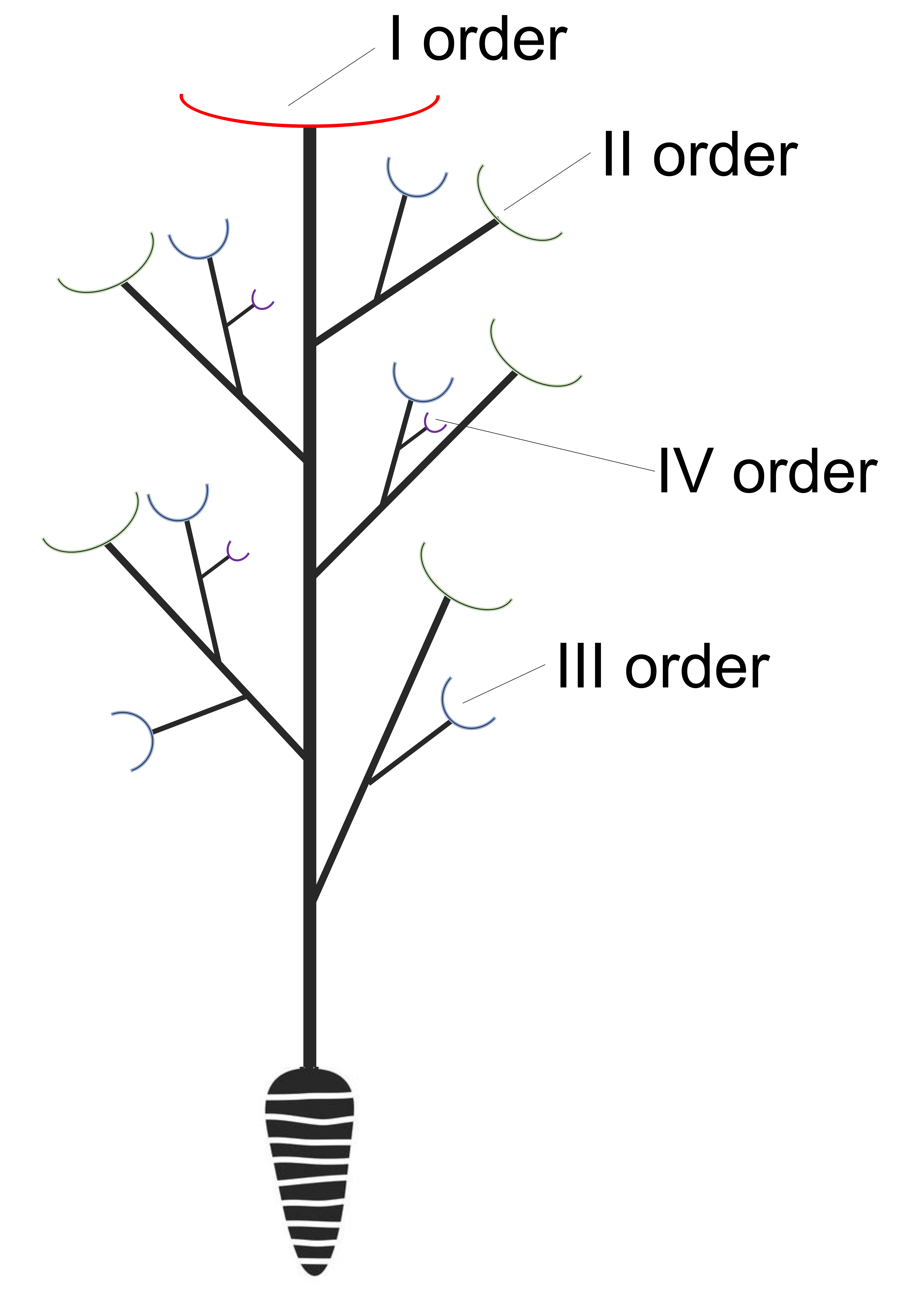

Supplement: Supplementary file 1 [file ijms-22-09713-s001.zip › Figure S1.jpg]
